# Impact of rumen-protected glucose on performance, milk composition, and selected blood metabolites of early lactating Holstein Friesian cows

**DOI:** 10.3389/fvets.2024.1498357

**Published:** 2024-12-18

**Authors:** Hairui Yu, Abdur Rahman, Hafeez Ur Rahman, Muhammad Khan, Maida Mushtaq, Guobo Quan, Muhammad Hammad Zafar, Zijian Li, Muhammad Aziz Ur Rahman

**Affiliations:** ^1^Key Laboratory of Biochemistry and Molecular Biology, Weifang Key Laboratory of Coho Salmon Culturing Facility Engineering, Institute of Modern Facility Fisheries, College of Biology and Oceanography, Weifang University, Weifang, China; ^2^Department of Animal Sciences, University of Veterinary and Animal Sciences, Lahore, Pakistan; ^3^Yunnan Animal Science and Veterinary Institute, Kunming, Yunnan Province, China; ^4^College of Animal Science and Technology, Yangzhou University, Yangzhou, Jiangsu, China; ^5^Institute of Dairy and Animal Sciences, University of Agriculture, Faisalabad, Pakistan

**Keywords:** cow, blood glucose, milk yield, milk lactose, non-esterified fatty acids

## Abstract

**Introduction:**

High-producing dairy cows often face calving stress and reduced feed intake during the transition period, leading to body fat mobilization to meet production demands. Supplementing rations with energy-dense sources like rumen-protected glucose (RPG) may enhance production performance in early lactation.

**Methods:**

This study evaluated the effects of RPG supplementation on feed intake, body condition score (BCS), production performance, and blood metabolites in 32 early-lactation Holstein Friesian cows (6 ± 1 DIM; milk yield: 30 ± 5 kg/day; body weight: 550 ± 50 kg; BCS: 3.00 ± 0.25). Cows were assigned to four groups (*n* = 8/group) and fed a basal diet (Control) or supplemented with 150 g (S-150), 300 g (S-300), or 450 g (S-450) of RPG for a 42-day trial after 2 weeks of adaptation.

**Results:**

Results showed significant improvements (*p* < 0.05) in final body weight, milk yield, energy-corrected milk, and milk-to-feed ratio with RPG supplementation, with the highest effects observed at 450 g/day. Milk components, including solids-not-fat, lactose, and total solids, also increased significantly. While feed intake remained similar (*p* > 0.05), blood glucose levels rose, and non-esterified fatty acids and *β*-hydroxybutyric acid concentrations decreased (*p* < 0.05), indicating reduced ketosis and negative energy balance.

**Conclusion:**

These findings suggest RPG supplementation at 450 g/day improves milk production, quality, and metabolic health in early lactating cows, warranting further exploration of higher dosages like 500–550 g/day.

## 1 Introduction

During the transition period, dairy cows experience an imbalance in bioenergetics due to the higher energy demands for milk synthesis and maintenance, which exceed dietary intake, resulting in a negative energy balance (NEB) (1). This imbalance occurs when the dietary supply of gluconeogenic precursors fails to meet the liver’s glucose output required by the mammary glands for milk production (2). Consequently, the body begins to catabolize amino acids from skeletal muscle and glycerol from adipose tissue to compensate for the energy deficit (3). Furthermore, reduced insulin activity due to NEB triggers lipolysis and the release of nonesterified fatty acids (NEFA) and beta-hydroxybutyric acid (BHBA) which are partially oxidized in the liver to produce ketone bodies (4). This process spares glucose for milk synthesis by inducing insulin resistance in muscle and adipose tissues (5). These metabolic adaptations, including glucose-sparing mechanisms, arise from the temporal mismatch of NEB and the compromised support for milk production. This energy deficiency increases susceptibility to metabolic disorders, including ketosis, fatty liver, and displaced abomasum in transition dairy cows (6). Additionally, research has shown that NEB can lead to a higher incidence of inflammatory responses, such as mastitis and endometritis, which may result in infertility (7, 8).

Glucose supplementation is critically important during the transition period to facilitate maximal milk synthesis and support immune function (2, 4). Glucose serves as a precursor for milk lactose, which plays a key role in the osmoregulation of milk (9). Hepatic glucose circulates in the blood, maintaining glucose homeostasis within a narrow range to meet the needs of peripheral tissues, including the mammary glands, muscles, adipose tissue, and the central nervous system (10, 11). It is estimated that approximately 72 grams of glucose are required to synthesis 1 kg milk (12, 13), and under homeostatic control, glucose supply does not limit milk synthesis (14). However, during specific circumstances such as the transition period, immune activation, and heat stress, glucose supply may become a limiting factor for milk yield in dairy cows (2). Moreover, it has been observed that fresh cows also experience inflammatory responses due to the ongoing uterine involution period (15). Therefore, providing a dietary glucose source with minimal rumen fermentation, such as rumen-protected glucose (RPG), represents a safer and more effective nutritional strategy to enhance intestinal glucose uptake, supporting higher milk production and postpartum immune function in dairy cows (16). Dietary supplementation of RPG may provide a surge of circulating glucose to support the milk production of high-producing animals. Studies revealed that RPG had a mixed impact on feed intake and milk production of dairy cows (17, 18). These variations in results might be due to varying precursors of glucose in products (mostly sucrose), dosage, and coating technology (freeze-drying or simple melting techniques). We hypothesized that dietary RPG supplementation could optimize lactating dairy cows’ production performance and health. The objectives of this experiment were to evaluate the influence of RPG levels on feed intake, production performance, milk quality parameters and selected circulating blood biomarkers in early lactating Holstein Friesian.

## 2 Materials and methods

### 2.1 Experimental site, research design, and farming practices

The protocols and procedures of this experiment were approved by the Animal Use and Care Committee of the University of Veterinary and Animal Science, Lahore, before the research trial (DAS/536: 12–02–2020). The trial was conducted at a commercial dairy farm (Nawab Dairy Farm, Khan Garh, Muzaffargarh; 30°4′10″N 71°11′39″E) to evaluate the influence of dietary supplementation with rumen-protected glucose (RPG) on production performance, milk quality, and health in early lactating cows. Thirty-two Holstein Friesian cows in early lactation (6 ± 1 days in milk; milk yield averaging 30 ± 5.0 kg/day; live body weight 550 ± 50 kg; and body condition score 3.00 ± 0.25) were randomized into four groups (*n* = 8 cows/group) and assigned to one of four RPG levels in a completely randomized design. The groups were fed a basal diet (Control) or a basal diet supplemented with 150 g (S-150), 300 g (S-300), or 450 g (S-450) of RPG. Menoglu-plus (containing 49% edible dextrose coated with 51% palm oil) was used as the RPG source (Menon, Shanghai, China). The product’s glucose content was determined using the manufacturer’s information, which included 90% rumen bypass and 90% intestine release rate, and the dosage was adjusted to assure accurate glucose supplementation for each group. The maximum dose was included after considering for the incidence of insulin resistance. The RPG supplementation was top-dressed to the basal diet of the respective group with receptive dosage. The basal diet was a total mixed ration and its detailed formulation and chemical compositions are provided in Tables 1, 2 respectively. The analysis of dietary ingredients and the basal diet was performed for dry matter, crude protein (method 984.13, N × 6.25; Kjeldahl method), and ether extract (method 920.39) following the official procedures of Baur and Ensminger (19). Neutral detergent fiber (*α*-amylase + sodium sulfite-treated filtration) and acid detergent fiber (sulfuric acid + cetyltrimethylammonium bromide-treated filtration) were analyzed sequentially using the Ankom-2000 fiber analyzer (Ankom Technology Corp., Fairport, NY, United States). After a two-week dietary adaptation period, a 40-day feeding trial was conducted. All cows were housed in a free-stall system equipped with ventilation fans. Cows were fed twice daily (6:00 and 18:00 h) with the basal diet mixed as a total mixed ration in a wagon mixer (Dunker T1-80, Storti SPA, Italy). Sand bedding was provided in stalls, plowed daily in the morning, and replaced as needed. Manure was scraped twice daily (4:00 and 19:00 h) using pressure water and a manual disc. Cows were milked twice daily (7:00 and 19:00 h) in the milking parlor using an automatic milking system. After milking, udders were washed, cleaned, and disinfected, with teats dipped following standard operating procedures.

**Table 1:** Basal diet feed formulation on a dry basis.

List	Inclusion level
Corn silage (%)	32.8
Rhodes grass hay (%)	7.0
Alfalfa haylage (%)	2.5
Wheat straw (%)	1.89
Corn grain (%)	24.0
Soybean meal (%)	12.5
Canola Meal (%)	5.5
Rapeseed meal (%)	3.2
Corn gluten meal 60% (%)	2.5
Sugarcane molasses (%)	4.0
Calcium carbonate (%)	0.6
Di-calcium phosphate (%)	0.2
Sodium bicarbonate (%)	0.5
Sodium chloride (%)	0.2
Mineral Mixture^1^ (%)	0.7
Urea (%)	0.208
Bypass fat-84 (%)	1.63
Toxin binder (clay base) (%)	0.052
Live Yeast (20 billion CFU/g) (%)	0.02

1 Vitamin & Premix mix contained 9.50% Ca, 10.20% P, 1.43% Mg, 2.50% K, 2.40% S, 4.00% Na, 6.90% Cl, 0.60% Fe, 1.80% Zn, 0.15% Cu, 0.17% Mn, 0.005% Se, 0.002% Co, 0.02% I, 800 IU/g of vitamin A, 200 IU/g of vitamin D, and 12.40 IU/g of vitamin E.

**Table 2:** Basal diet compositional characteristics on a dry basis.

Nutrient composition	
Variable	Value
Dry matter (%)	53.21
Crude protein (%)	17.48
Neutral detergent fiber (%)	28.41
Acid detergent fiber (%)	15.89
Non-fibrous carbohydrates (%)	45.24
Ether extract (%)	4.54
Predicted values^7^	
Metabolizable protein (g/kg of DM)	121.0
RUP^1^ (% CP)	41.01
RDP^2^ (% CP)	58.99
ME^3^ (Mcal/kg of DM)	2.84
NEL^4^ (Mcal/kg of DM)	1.79
DCAD^5^ (mEq/kg)	209.0

1 RUP, rumen undegradable protein.

2 RDP, rumen degradable protein.

3 ME, metabolizable energy.

4 NEL, net energy for lactation.

5 DCAD, dietary cation-anion difference.

7 predicted values were calculated by NDS-RUMIN software.

### 2.2 Body weight and feed intake

Cows’ live body weights were recorded before the experiment and then weighted weekly using a digital weighing balance to estimate the changes in body weight. Feed was offered twice (6:00 and 18:00 h) a day and orts were recorded the next day before morning feeding to calculate the dry matter intake (DMI). The DMI was calculated using the following Equation 1.


(1) $$\begin{array}{l} DMI\;\left(\mathrm{kg}\right)=\left(\left(\mathrm{Feed offer}\;\left(\mathrm{kg}\right)-\mathrm{orts}\;\left(\mathrm{kg}\right)\;X\;DM\;\mathrm{of}\;\mathrm{feed}\right)\right)/\\ \mathrm{number}\;\mathrm{of}\;\mathrm{cows}\;\mathrm{in}\;\mathrm{group} \end{array}$$

### 2.3 Milk volume and compositional characteristics

The milk volume of each cow was recorded daily using a digital weighing balance. Following a sterile protocol, approximately 30 and 20% of the total milk volume were sampled in the morning and evening, respectively. The collected samples were composited, and each cow’s subsample was taken daily to create a representative sample. These samples were then analyzed for milk composition using a Lactoscan milk analyzer (Lac-toscan Farm Eco, Milkrotronic, Nova Zagora, Bulgaria). Energy-corrected milk, fat-protein-corrected milk, and the milk-to-feed ratio were calculated using Equations 2–4, respectively.


(2) $$ECM\;\left(\mathrm{kg}\right)=\left(\begin{array}{l} \left(0.325\;X\;\mathrm{milk yield}\;\left(\mathrm{kg}\right)\right)+\\ \left(12.86\;X\;\mathrm{milk}\;\mathrm{fat}\;\mathrm{yield}\;\left(\mathrm{kg}\right)\right)+\\ \left(7.04\;X\;\mathrm{milk}\;TP\;\mathrm{yield}\;\left(\mathrm{kg}\right)\right) \end{array}\right)$$

(3) $$\begin{array}{l} \mathrm{FPCM}\;\left(\mathrm{kg}\right)=\left(0.4\;X\;\mathrm{Milk Yield}\;\left(\mathrm{kg}\right)\right)+\\ \left(10\;X\;\mathrm{Fat}\;\mathrm{Yield}\;\left(\mathrm{kg}\right)\right)+\\ \left(7\;X\;\mathrm{Protein Yield}\;\left(\mathrm{kg}\right)\right) \end{array}$$

(4) $$\mathrm{Milk to Feed ratio}=\left(ECM\;\left(\mathrm{kg}\right)/DMI\;\left(\mathrm{kg}\right)\right)$$

### 2.4 Blood biochemical analysis

Weekly blood samples of all cows were collected from their coccygeal vein 4 h after morning feeding by following a sterile method. Blood samples were collected in non-EDTA-containing vacutainers, and serum was separated by centrifugation at 2,000 × g for 15 min. The harvested serum samples were stored in labeled tubes at −20°C until further analysis. The serum was analyzed for BHBA, NEFA, and glucose, using commercially available enzymatic kits (Biosystems, Barcelona, Spain) by following the manufacturer’s instructions.

### 2.5 Statistical analysis

The collected data were subjected to QQ plots and log transformation (SAS v. 9.4, University Edition, SAS In-stitute Inc., Cary, NC, United States) to test for normality and find the missing values. Data on production performance were analyzed using the MIXED procedure of SAS (SAS v. 9.4, University Edition, SAS Institute Inc., Cary, NC, United States). Individual cows were considered as the experimental units. The model included the following fixed effects: treatments, weeks, and the interaction of treatments × weeks. The statistical model was:


Yijkl=μ+Tj+Wk+TWjk+εijkl,

where, Yijkl = observation, μ = population mean, Tj = treatment effect, Wk = Weeks effect, TWjk = interaction of treatment and Weeks, εijkl = residual error. For multiple observations across weeks, the autoregressive [AR (1)] structure was applied within the framework of the repeated measures based on Akaike information criterion values. Blood metabolites were analyzed using GraphPad Prism (version 9.0). Significance was declared at *p* < 0.05, and means were compared using Tukey’s test. Weekly raw data values of milk yield were subjected to broken line regression using GraphPad Prism (version 9.0) for dosage-to-yield curve fit analysis with the following model.


$$Y=L+U\times \left(R-X\right)\times 1$$

Where: Y = dependent variable, L = theoretical maximum, R = requirement, X = independent variable, I = 1 (if X < R) or I = 0 (if X > R), U = rate constant.

## 3 Results

### 3.1 Production performance

**Figure 1:**
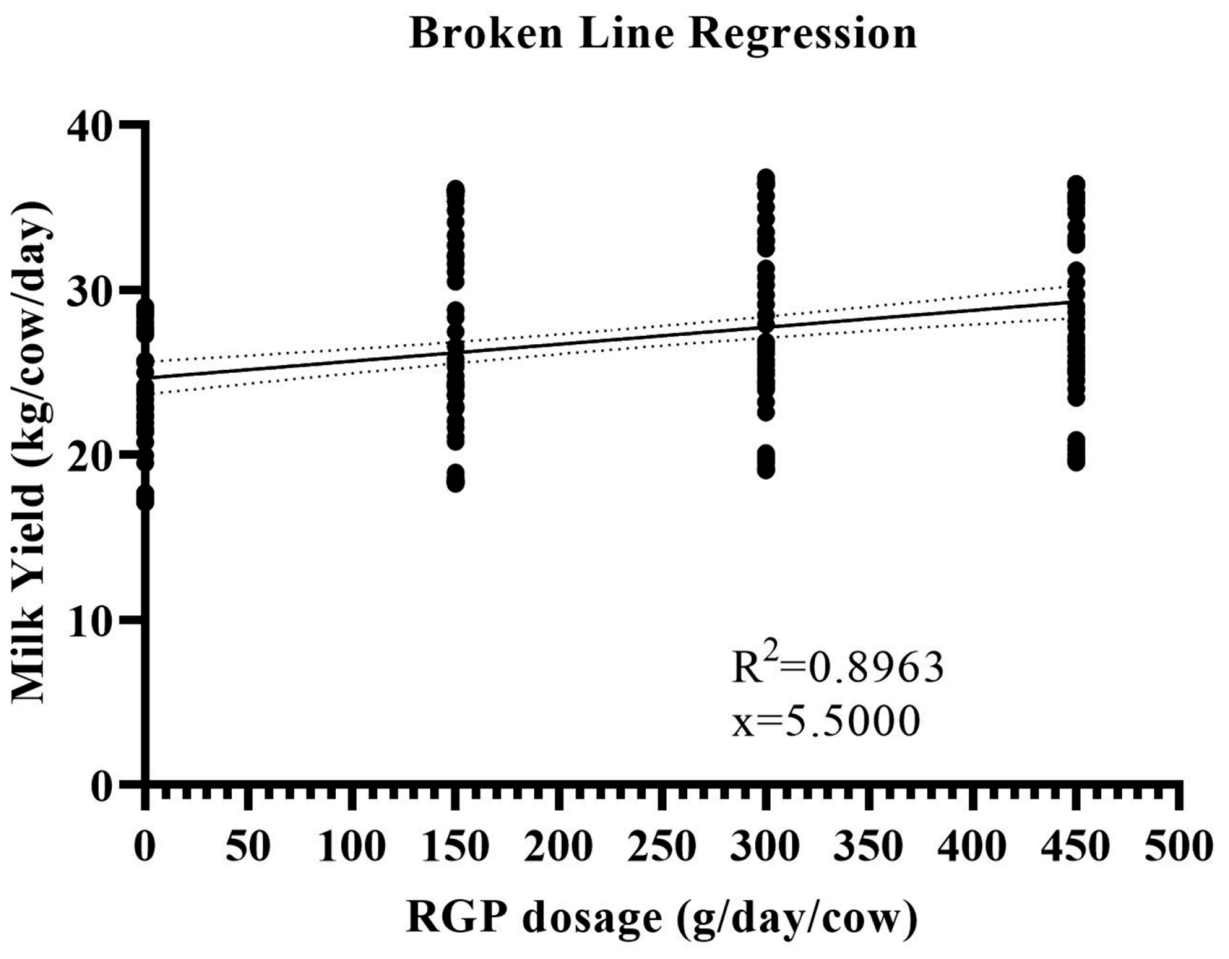
Dose–response (0–450 g/day/cow) broken line analysis to estimate the curve fit on weekly milk yield (kg/cow/day). The results were significant (*p* < 0.005) with the equation Y = 0.01026*X + 24.64 and, R^2^ = 0.08963.

According to Table 3, dietary supplementation with RPG significantly influenced the production performance parameters of early lactating cows. Final body weight increased linearly with RPG supplementation; with cows receiving 450 g/day RPG showing the greatest (*p* < 0.05) increase across the groups. Similarly, milk yield, energy-corrected milk, and fat-protein-corrected milk were significantly greater (*p* < 0.05) in the supplemented groups compared to cows fed the control diet. There was a significant treatment-by-week interaction for these milk production parameters, indicating that milk yield improved with increasing days in milk (*p* < 0.05). The milk-to-feed ratio of early lactating cows also improved significantly (*p* < 0.05) with RPG supplementation, while feed intake remained similar (*p* > 0.05) across the groups. The broken line analysis revealed that dietary dosage of RPG @ 450 improved weekly milk yield (*p* < 0.05), suggesting a dire need to explore the upper levels like 500 and 550 g/cow/day (Figure 1).

**Table 3:** Production performance of early lactating cows supplemented with different levels of rumen-protected glucose.

Variable	Treatments^1^				SEM^2^	*p*-value		
	Control	S-150	S-300	S-450		Trt^3^	week	Trt*week
FBW (kg/cow)	478.8^d^	494.2^bc^	501.4^bc^	519.2^a^	4.628	<0.0001	–	–
Feed intake (Kg/d)	27.07	29.28	28.81	28.93	3.246	0.125	0.131	0.151
Milk Yield (Kg/d)	23.88^d^	27.05^c^	28.23^ab^	28.62^ab^	0.196	<0.0001	<0.0001	<0.0001
ECM (kg/d)	21.48^d^	22.93^c^	23.16^ab^	23.27^ab^	0.137	<0.0001	<0.0001	0.010
FPCM (Kg/d)	21.07^d^	22.68^c^	22.96^b^	23.16^a^	0.120	<0.0001	<0.0001	<0.0001
Milk to feed ratio	0.76^d^	0.81^b^	0.80^c^	0.82^a^	0.005	<0.0001	<0.0001	<0.0001

1 treatments = basal diet without supplementation (control), and basal diets with supplementation @ 150 g (S-150), 300 g (S-300), and 450 g (S-450) of rumen-protected glucose per day per cow.

2 SEM, standard error of means.

3 Trt, represents *p*-value for treatments.

The superscripts a–d without common sharing in a row mean a significant difference (*p* < 0.05), while common sharing among groups represents a non-significant difference (*p* > 0.05).

### 3.2 Milk compositional characteristics

The milk protein and milk fat contents of cows were not significantly influenced (*p* > 0.05) by dietary supplementation with RPG (Table 4). However, milk lactose, solids-not-fat, and total solids contents were significantly improved (*p* < 0.05) in the RPG-supplemented groups compared to the control group. There was also a significant treatment-by-week interaction between milk lactose and solids-not-fat contents. Milk lactose content was highest in cows supplemented with 450 g/day RPG, followed by 300 g/day RPG (*p* < 0.05). There was no significant difference in milk lactose content between the control and 150 g/day RPG-supplemented groups (*p* > 0.05). Solids-not-fat content increased linearly with increasing RPG supplementation (*p* < 0.05). Similarly, total solids contents were greater in the S-300 and S-450 supplemented groups compared to the other treatments (*p* < 0.05).

**Table 4:** Milk Composition of early lactating cows supplemented with different levels of rumen-protected glucose.

Variable	Treatments				SEM	*p*-value		
	Control	S-150	S-300	S-450		Trt	Week	Trt*week
Milk protein (%)	2.99	3.05	2.94	2.99	0.031	0.089	0.444	0.864
Milk fat (%)	4.10	4.25	4.22	4.21	0.052	0.207	0.025	0.906
Milk lactose (%)	4.33^cd^	4.33^cd^	4.36^b^	4.37^a^	0.009	0.011	0.245	0.002
Milk solid not fat (%)	7.87^d^	7.90^c^	7.94^b^	7.95^a^	0.016	0.003	0.506	0.045
Milk total solids (%)	11.98^d^	12.15^c^	12.17^ab^	12.17^ab^	0.055	0.044	0.022	0.634

The superscripts a-d without common sharing in a row mean a significant difference (*p* < 0.05), while common sharing among groups represents a non-significant difference (*p* > 0.05).

### 3.3 Blood biochemical analysis

**Figure 2:**
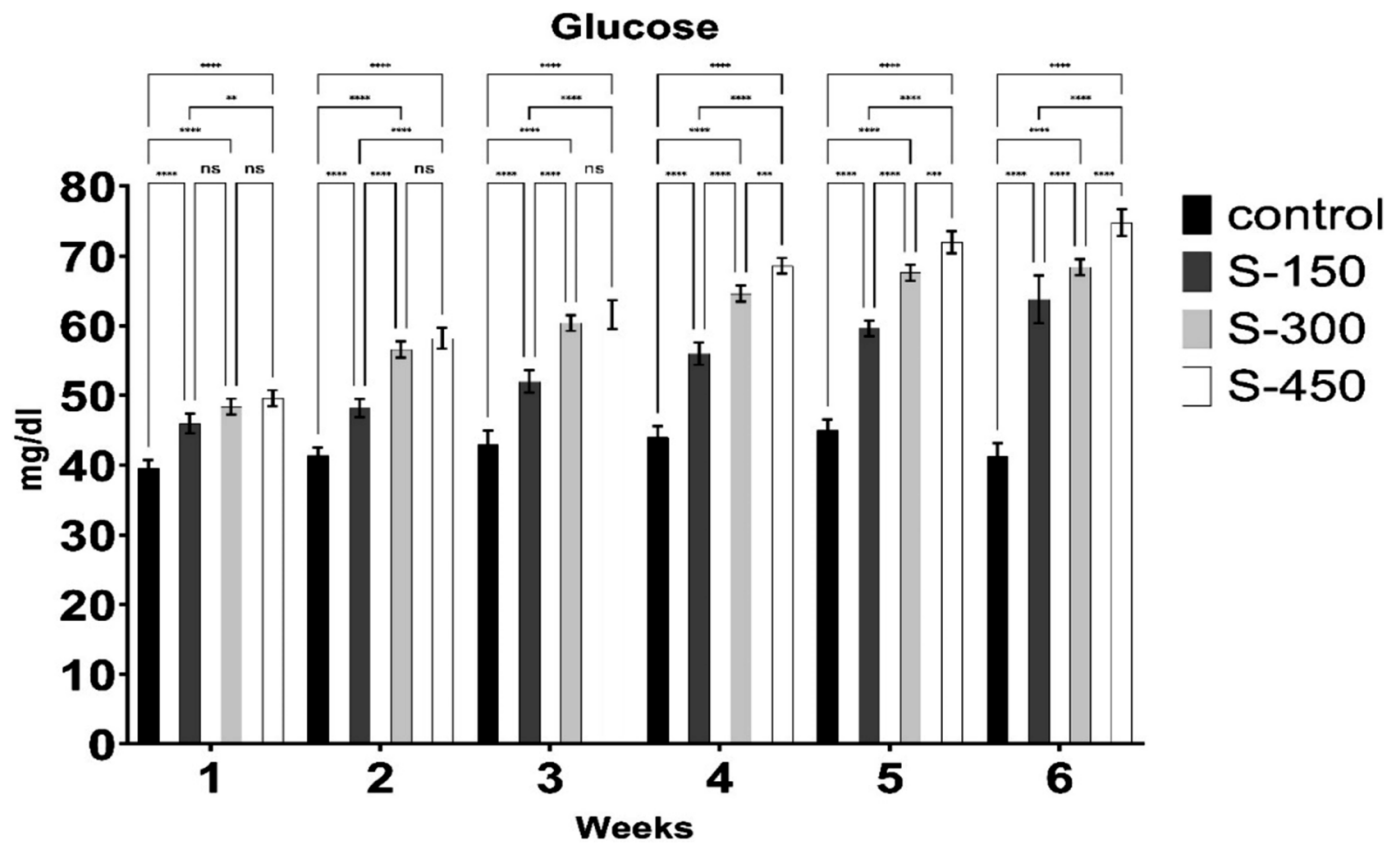
Weekly blood glucose concentration (mg/dl) of early lactating cows supplemented with different rumen-protected glucose (basal diet without supplementation (control); and with 150 (S-150), 300 (S-300), and 450 (S-450) g/d/cow, rumen-protected glucose supplementation). ***donated to significant difference (*p* < 0.05) while ns means non-significant difference (*p* > 0.05) across different groups.

**Figure 3:**
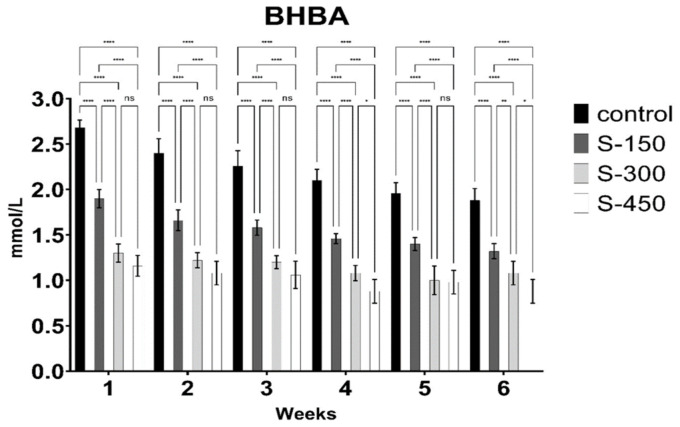
Weekly *β*-hydroxybutyric acid (BHBA) concentration (mmol/L) of early lactating cows supplemented with different rumen-protected glucose (basal diet without supplementation (control); and with 150 (S-150), 300 (S-300), and 450 (S-450) g/d/cow, rumen-protected glucose supplementation). ***donated to significant difference (*p* < 0.05) while ns means non-significant difference (*p* > 0.05) across different groups.

**Figure 4:**
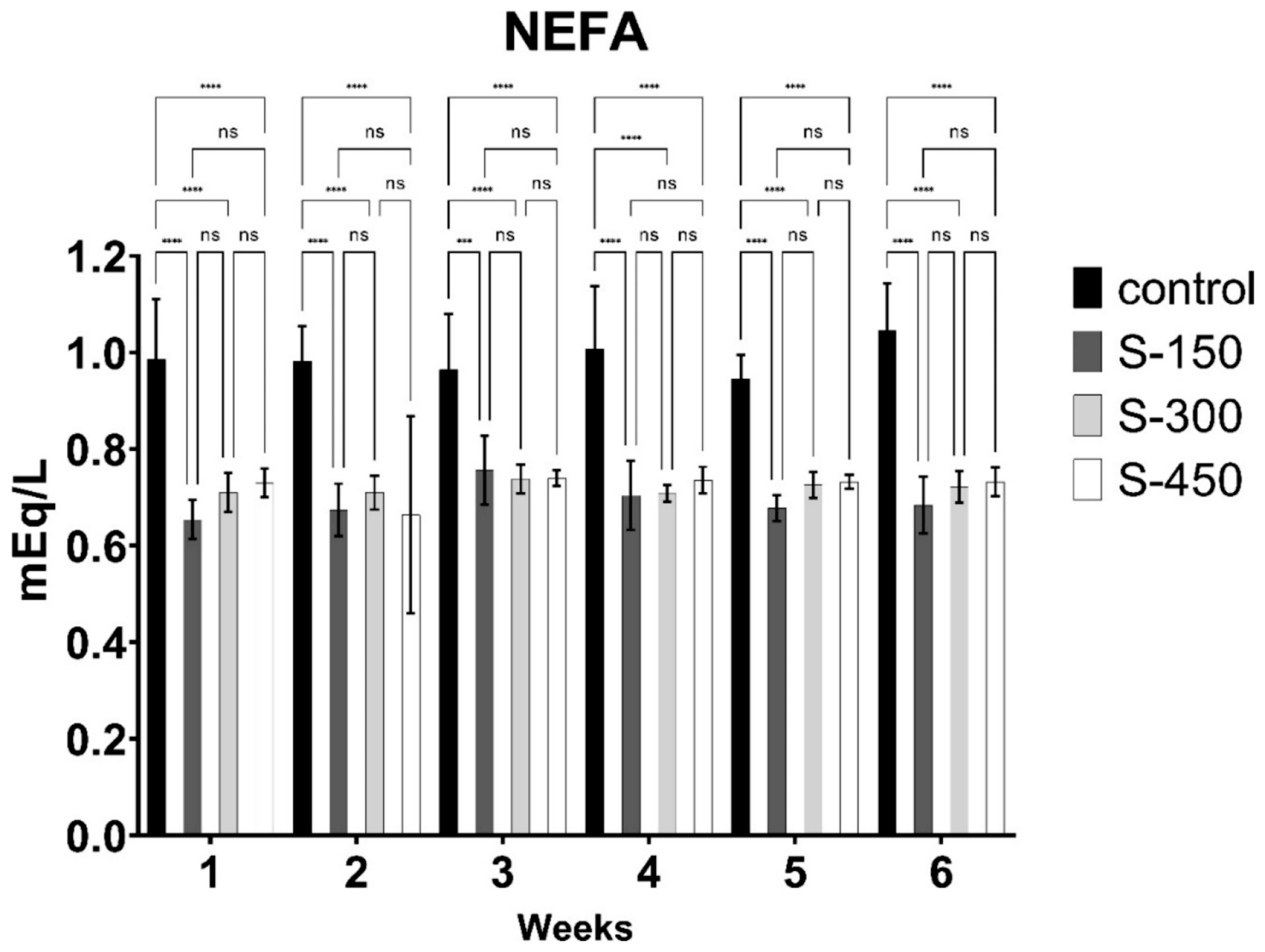
Weekly non-esterified fatty acids (NEFA) concentration (mEq/L) of early lactating cows supplemented with different rumen-protected glucose (basal diet without supplementation (control); and with 150 (S-150), 300 (S-300), and 450 (S-450) g/d/cow, rumen-protected glucose supplementation). ***donated to significant difference (*p* < 0.05) while ns means non-significant difference (*p* > 0.05) across different groups.

The blood glucose, NEFA, and BHBA concentrations of dairy cows were significantly influenced (*p* < 0.05) by dietary supplementation with RPG (Figures 2–4). Blood glucose concentration increased linearly with RPG supplementation (Figure 2). The highest blood glucose concentration was observed in the S-450 group, followed by the S-300 and S-150 groups, with the control group having the lowest levels (*p* < 0.05). However, during the first 3 weeks, blood glucose concentrations were similar (*p* > 0.05) between the S-300 and S-450 groups. BHBA concentration decreased linearly (*p* < 0.05) with RPG supplementation. Across all weeks, the lowest BHBA concentration was found in the S-450 group, followed by the S-300 and S-150 groups, with the control group having the highest levels (Figure 3). Similarly, NEFA concentration decreased significantly (*p* < 0.05) from week 1 to week 6 in the RPG-supplemented groups compared to the control group (Figure 4). However, the RPG-supplemented groups’ differences were insignificant (*p* > 0.05).

## 4 Discussion

During early lactation, dairy cows, require more glucose supply to meet the lactose synthesis and milk production requirements (11). In addition, a higher dietary supply of glucose can combat parturition stress and help mitigate negative energy balance to smoothen the production cycle (2). For this, mostly glucose precursors such as propylene glycol are supplemented by dairy farmers (20, 21). However, these precursors undergo two-step biological conversion (first in the rumen and then in the liver); thus, it impacts the efficiency of bioenergetics of high-producing cows (22). In the recent past, usage of rumen-protected glucose particularly during the early lactating phase has been observed (16). The rumen-protected glucose bypasses the rumen and ensures the glucose in the intestine for hepatic metabolism and availability for milk production in a short time (16). Moreover, RPG supplementation might alter rumen fermentation patterns, potentially impacting methane emissions. Thus, in this way it could improve feed efficiency and energy metabolism, leading to reduced methane per unit of milk. Keeping all this in view, the current study aimed to investigate the influence of RPG on production performance and health biomarkers of early lactating Holstein Frisian cows. In this study, the dry matter intake was similar across the groups. These results are consistent with McCarthy, Dooley (16) as they also reported that RPG dietary supplementation did not change dairy cows’ dry matter intake. However, contrary to the present study, Omphalius, Lemosquet (23) reported increased dry matter intake of those early lactating dairy cows exposed to post-ruminal glucose infusion. The non-significant difference in dry matter intake might be because of an increase in blood glucose concentration which might suppress the hunger reflex (24) as it is well established that dietary starch enrichment resulted in a satiety impact on the DMI of dairy cows by raising blood glucose concentration (25). In our study, dietary supplementation of RPG increased milk production linearly suggesting higher glucose avail-ability for milk production. These results can be associated with the glucose-sparing mechanism as RPG ensures more glucose uptake by mammary glands for lactose synthesis and milk production (26). Onetti and Grummer reported that RPG supplementation improved the milk yield of dairy cows. However, contrary to our results, McCarthy, Dooley (16) reported that dietary supplementation of RPG did not improve the milk yield during the early lactation phase. The difference in results might be due to differences in the dosage of supplementation which was only 6% on a dry basis. The milk-to-feed ratio is an important indicator that helps in forecasting dairy farming profitability (27). In the present study, dietary supplementation of RPG improved the milk-to-feed ratio suggesting improved economic efficiency and profitability in dairy farming. In the present study, milk compositional characteristics such as lactose, solid not fat, and total solids contents were influenced by dietary supplementation of RPG. These results contradict the findings of McCarthy, Dooley (16) as they found no changes in milk lactose contents of the RPG supplemented group. The difference in results can be associated with palm oil coating technology as Hammon, Metges (28) found increased total solids, lactose, and solid not fat contents of those dairy cows supplemented with bypass fat. These results can be associated with the availability of blood glucose for lactose synthesis by mammary glands. It is well established that glucose is a precursor for lactose, higher dietary supply of glucose precursors resulted in raised blood glucose concentration and ultimately lactose contents and milk yield of dairy cows (2, 29). However, the dietary supply of RPG in the current study did not affect milk protein and fat contents. These results align with McCarthy, Dooley (16) as they reported that feeding 6% RPG in the postpartum period did not influence the milk fat and protein contents of dairy cows.

The blood metabolites are well-known biomarkers directly associated with dairy cows’ health (30). The present study showed that dietary supplementation of RPG resulted in increased glucose concentration, and decreased the NEFA and BHBA concentrations in the blood of dairy cows. A previous study by McCarthy, Dooley (16) also reported increased blood glucose levels in dairy cows by feeding RPG. These results can be associated with more availability of RPG at the tissue level for metabolism because of the bypass mechanism. The greater glucose availability augments the above finding regarding the higher milk lactose contents and milk Yield. It is well-established that blood glucose is driven by mammary glands for lactose synthesis (2, 29), and therefore it is commonly regarded as a limiting factor in milk production. Lower blood glucose levels because of dietary imbalance to meet the production requirements of high-producing dairy animals led to NEB, and higher mobilization of body reserves (primarily fat and glycogen) resulting in greater production of ketone bodies (like BHBA and NEFA) than normal ranges. NEFA and BHBA are products of fat metabolism in the liver, get oxidized, and result in ketone bodies which causes ketosis (30). Therefore, these are biomarkers to access the NEB in dairy cows, particularly during the early lactating phase. In the present study, blood concentrations of NEFA and BHBA were lower in treatment groups compared with control groups suggesting better bioenergetics with RPG supplementation. These results are per McCarthy, Dooley (16) as they also found lower levels of NEFA and BHBA in RPG-supplemented groups of the cows. These results can be associated with better bioenergetics (Glucose-sparing impact of RPG) to meet dairy cows’ milk production requirements suggesting, that RPG supplementation can decrease the incidence of negative energy balance and ketosis.

## 5 Conclusion

Based on results, dietary supplementation of rumen-protected glucose @ 450 g/d/cow, increased the milk yield of early lactating dairy cows with better milk quality by improving milk lactose, and total solids contents. In addition, blood biomarkers such as glucose, NEFA, and *β*-hydroxybutyrate suggest that rumen-protected glucose supplementation smoothens the health of early lactating cows by augmenting the bioenergetics and mitigating the negative energy balance. The broken-line regression analysis indicated the need to explore additional levels of rumen-protected glucose supplementation in high-producing animals to optimize performance outcomes. However, the potential for higher RPG dosages to induce insulin resistance presents a notable limitation of the current study. Consequently, further research is necessary to investigate the effects of elevated RPG supplementation levels and to elucidate the underlying mechanisms associated with insulin resistance.

## Data Availability

The original contributions presented in the study are included in the article/supplementary material, further inquiries can be directed to the corresponding authors.
